# Supplementary material for: Multi-Analytical Insight into the Non-Volatile Phytochemical Composition of Coleus aromaticus (Roxb.) Benth
Source: Metabolites. 2025 Dec 23;16(1):15. doi: 10.3390/metabo16010015 (PMC12844460; doi:10.3390/metabo16010015)
Supplement: Supplementary file 1 [file metabolites-16-00015-s001.zip › metabolites-4041555-supplementary.pdf]

metabolites

MDPI

Supplementary Materials

A Multi-Analytical Insight into the Non-Volatile Phytochemical Composition of *Coleus aromaticus* (Roxb.) Benth.

Chiara Toniolo[1]*, Martina Bortolami [1], Adriano Patriarca [2], Daniela De Vita [1], Fabio Sciubba [1,3,4], and Luca Santi [1]

[1] Department of Environmental Biology, Sapienza University of Rome, Piazzale Aldo Moro 5, 00185 Rome, Italy; chiara.toniolo@uniroma1.it (C.T.), martina.bortolami@uniroma1.it (M.B.); daniela.devita@uniroma1.it (D.D.V.), fabio.sciubba@uniroma1.it (F.S.), l.santi@uniroma1.it (L.S.)

[2] Department of Chemistry, University of Rome Sapienza, Piazzale Aldo Moro 5, 00185 Rome, Italy; adriano.patriarca@uniroma1.it (A.P.)

[3] NMR-Based Metabolomics Laboratory (NMLab), Sapienza University of Rome, Piazzale Aldo Moro 5, 00185 Rome, Italy

[4] Interdepartmental Center of Applied Sciences for the Protection of the Environment and Cultural Heritage (CIABC), Sapienza University of Rome, Piazzale Aldo Moro 5, 00185 Rome, Italy

* Correspondence: chiara.toniolo@uniroma1.it; +39 06 49912195

List of Reagents, Solvents, and Standards

A complete list of all chemical reagents and standards used is provided here for reference.

Standards and solvents were purchased from Sigma (Sigma-Aldrich, USA). All chemicals and solvents were of analytical grade. The stationary phase used for HPTLC analysis consisted of silica gel 60 F254 precoated plates (20 × 10 cm), purchased from Merck (USA).

Solvents (analytical grade) used for sample and standard preparation, as well as for mobile phases in HPTLC analysis, included: ethyl acetate, dichloromethane, chloroform, methanol, 1-butanol, acetic acid, and formic acid.

Chemicals and reagents for NMR analysis: deuterium oxide (D_2O), 3-(trimethylsilyl)-propionic-2,2,3,3-d4 acid sodium salt (TSP), deuterated chloroform ($CDCl_3$) hexamethyldisiloxane (HMDSO).

HPTLC standards included the following:

- Amino acids: alanine, arginine, asparagine, aspartic acid, cysteine, glutamic acid, glutamine, glycine, histidine, isoleucine, leucine, lysine, methionine, phenylalanine, proline, serine, theanine, threonine, tryptophan, tyrosine, and valine.
- Organic acids and flavonoids: apigenin, caffeic acid, chlorogenic acid, cinnamic acid, gallic acid, luteolin, p-coumaric acid, quercetin, and rutin.

HPTLC derivatization agents included:

- For anisaldehyde-sulfuric acid reagent: anisaldehyde, sulfuric acid, acetic acid, and methanol.
- For Natural Product Reagent: phenylboronic acid and ethyl acetate.
- For ninhydrin reagent: ninhydrin, isopropanol, and acetic acid.

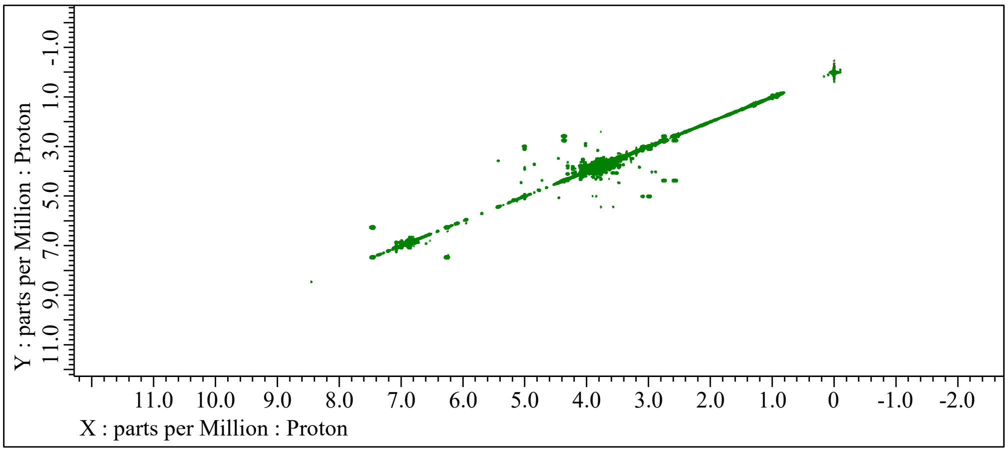

Supplementary Figure S1. ^{1}H-^{1}H TOCSY spectrum of *C. aromaticus* leaves of BD-HAP extract.

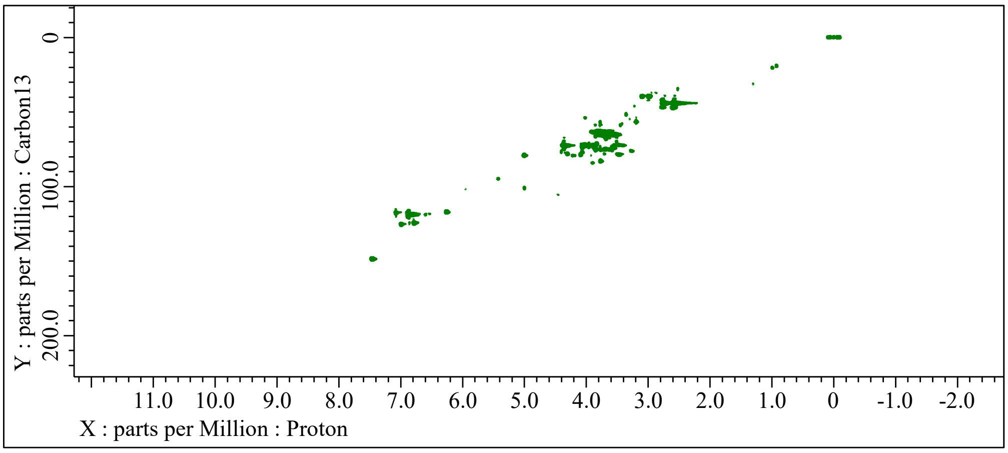

Supplementary Figure S2. ^{1}H-^{13}C HSQC spectrum of *C. aromaticus* leaves of BD-HAP extract.

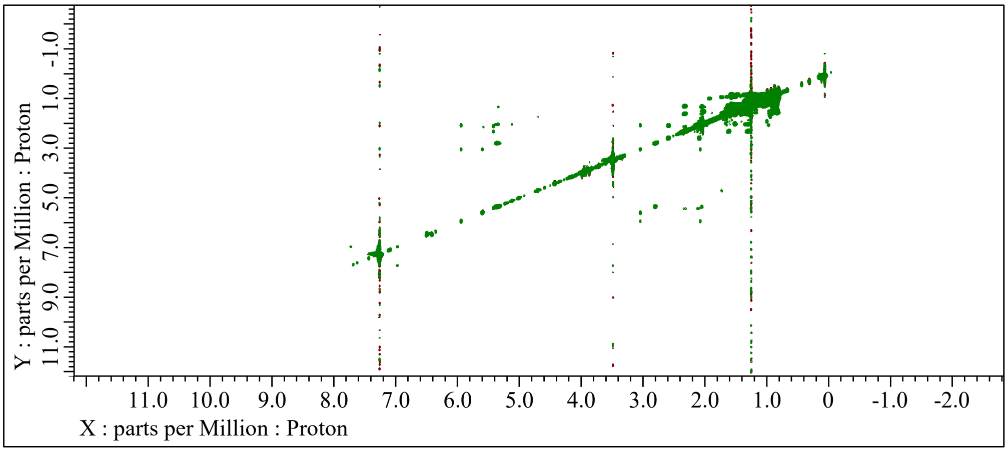

Supplementary Figure S3. ^{1}H-^{1}H TOCSY spectrum of *C. aromaticus* leaves of BD-lipophilic extract.

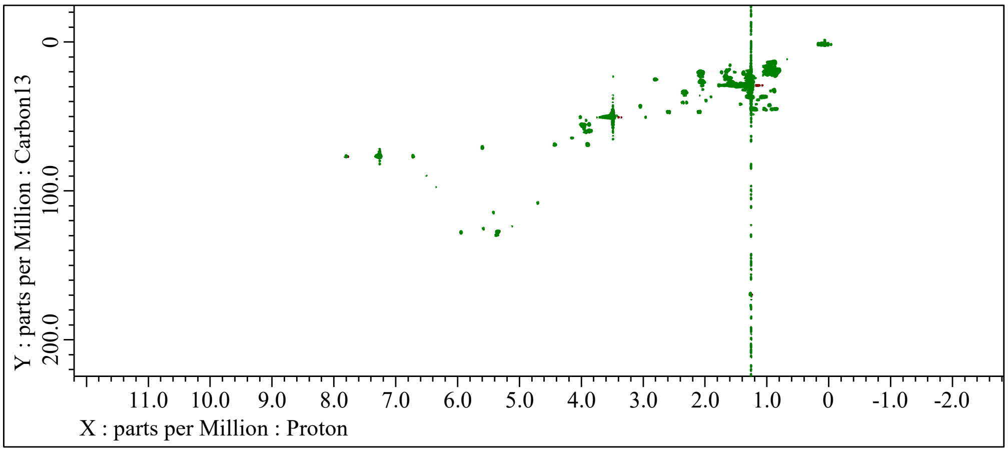

Supplementary Figure S4. ^{1}H-^{13}C HSQC spectrum of *C. aromaticus* leaves of BD-lipophilic extract.

Supplementary Table S1. ^{1}H and ^{13}C spectrum assignment and resonances of *C. aromaticus* leaves. Signal multiplicities: s: singlet, d: doublet, t: triplet, q: quartet, dd doublet of doublets, m: multiplet, bs: broad singlet. In bold are reported signal resonances chosen for quantification.

Compound	^{1}H δ ppm	Multiplicity	Assignment	^{13}C δ (ppm)
**Amino acids**				
**Alanine**	**1.48**	**d**	**β-CH$_3$**	**18.96**
	3.8	q	α-CH	53.27
**Asparagine**	**2.86**	**dd**	**β'-CH**	**37.27**
	2.89	dd	β-CH	37.27
	4.01	m	α-CH	54.02
**γ-Aminobutyric acid (GABA)**	3.01	t	y-CH$_2$	41.97
	2.28	t	α-CH$_2$	37.32
	**1.89**	**m**	**β-CH$_2$**	**26.33**
**Glutamic acid**	2.04	**m**	y-CH$_2$	29.68
	**2.36**	m	**β-CH$_2$**	**36.52**
	3.74	m	α-CH	57.3
**Glutamine**	**2.14**	**m**	**y-CH$_2$**	**28.98**
	2.45	m	β-CH$_2$	33.72
	3.81	m	α-CH	56.88
**Threonine**	**1.32**	**d**	**g-CH$_3$**	**22.3**
	3.6	m	α-CH	63.15
	4.27	m	β-CH	68.81
**Organic Acids**				
**Acetic Acid**	**1.92**	**s**	**CH$_3$**	**25.99**
**Caffeic Acid**	**6.3**	**d**	**CH-5**	**124.33**
	7.32	d	CH-4	144.2
	6.99	dd	CH-3	119
	7.21	d	CH-1	117.22
	7.09	d	CH-2	124.12

Citric Acid	2.85	d	y-CH_2	47.39
	3.01	d	α-CH_2	47.39
**Formic Acid**	**8.46**	**s**	**CH**	**143.64**
**Fumaric Acid**	**6.51**	**s**	**CH=CH**	**138.5**
**Malic Acid**	**4.28**	**dd**	**α-CH**	**72.93**
	2.38;2.69	dd	β,β'-CH	45.27
**Rosmarinic Acid**	**6.27**	**d**	**CH-1 Caff**	**114.72**
	7.57	d	CH-2 Caff	149.01
	6.88	dd	CH-3 Caff	122.18
	6.79	d	CH-4 Caff	116.61
	7.14	sd	CH-5 Caff	115.52
	6.79	dd	CH-1 3,4DPLA	123.48
	6.88	dd	CH-2 3,4DPLA	116.79
	7.07	sd	CH-3 3,4DPLA	117.87
**Carbohydrates and polyols**				
**Fructose**	3.69	m	**CH-1**	62.94
	\	\	**C-2**	**104.23**
	**4.22**	**d**	**CH-3**	**77.24**
	4.06	m	CH-4	75.04
	3.9	m	CH-5	83.37
**α-Glucose**	**5.23**	**d**	**CH-1**	**94.83**
	3.55	m	CH-2	63.3
	3.72	m	CH-3	73.89
	3.42	m	CH-4	70.59
	3.84	m	CH-5	72.64
	3.73,3.90	m	CH_2-6	75.26
**β-Glucose**	**4.69**	**d**	**CH-1**	**98.65**
	3.26	m	CH-2	62.07
	3.5	m	CH-3	78.84
	3.42	m	CH-4	70.77
	3.48	m	CH-5	74.43
	3.74, 3.91	m	CH_2-6	61.79
**Raffinose**	**5.45**	**d**	**GLC CH-1**	**102.4**
	5.01	d	GAL CH-1	93.9
	4.22	d	FRU CH-3	63.6
**Sucrose**	**5.44**	**d**	**G CH-1**	**93.22**
	3.59	m	CH-2	72.11
	3.79	m	CH-3	73.54
	3.48	m	CH-4	70.26
	3.85	m	CH-5	73.38
	3.82	m	CH_2-6	61.18
	3.69	m	F CH_2-1'	62.44
	\	\	C-2	104.85
	4.22	m	CH-3'	77.45

	4.06	m	CH-4’	75.04
	3.9	m	CH-5’	82.44
	3.82	m	CH_2-6	63.38
**Lipids & Sterols**				
**β-Sitosterol**	1.08, 1.85	m	CH_2-1	37.19
	1.51, 1.84	m	CH_2-2	31.5
	3.52	m	CHOH-3	71.81
	2.28	m	CH_2-4	42.37
	5.34	m	CH-6	121.79
	1.52, 1.98	m	CH_2-7	31.98
	1.46	m	CH-8	31.78
	0.99	m	CH-14	56.74
	1.57	m	CH_2-15	24.25
	1.26, 1.85	m	CH_2-16	28.37
	**0.68**	**s**	**CH_3-18**	**12.2**
	1.01	s	CH_3-25	19.12
**Campesterol**	1.08, 1.85	m	CH_2-1	37.19
	1.51, 1.84	m	CH_2-2	31.5
	3.52	m	CHOH-3	71.81
	2.28	m	CH_2-4	42.37
	5.34	m	CH-6	121.79
	1.52, 1.98	m	CH_2-7	31.98
	1.46	m	CH-8	31.78
	0.99	m	CH-14	56.74
	1.57	m	CH_2-15	24.25
	1.26, 1.85	m	CH_2-16	28.37
	**0.7**	**s**	**CH_3-18**	**12.21**
	1.01	s	CH_3-25	19.12
Glycerophospholipids	3.65-3,55	dd	CH_2	68.23
	4.05-4.15	dd	CH_2	68.23
	5.21	m	CH	77.46
**Monounsaturated ω-9 fatty acid (ω-9 FA)**	0.88	t	CH_3	14.1
	1.27	m	n-CH_2	29.33
	**2.03**	**m**	**CH_2-CH=CH**	**27.16**
**Polyunsaturated ω-6 fatty acid (ω-6 FA)**	0.86	t	CH_3	14.35
	1.36	m	n-CH_2	29.41
	2.04	m	$C\underline{H}_2$-CH=CH	29.5
	5.37	m	CH=CH	128.26, 130.5
	**2.76**	**t**	**=CH-$C\underline{H}_2$-CH=**	**26.83**
	2.06	m	$C\underline{H}_2$-CH_2-CO_2^-	24.57
	2.31	t	CH_2-CO_2^-	34.02
**Polyunsaturated ω-3 fatty acid (ω-3 FA)**	0.95	t	CH_3	14.27
	1.37	m	n-CH_2	29.22
	2.04	m	$C\underline{H}_2$-CH=CH	27.24

	5.36	m	CH=CH	130.45; 128.33
	**2.82**	**t**	**=CH-C<u>H</u>$_2$-CH=**	**26.89**
	2.03	m	C<u>H</u>$_2$-CH$_2$-CO$_2^-$	24.56
	2.3	t	CH$_2$-CO$_2^-$	33.94
**Saturated fatty acid (SFA)**	0.87	t	CH$_3$	14.1
	1.26	m	n-CH$_2$	29.33
	1.62	m	C<u>H</u>$_2$-CH$_2$-CO$_2^-$	24.57
	**2.3**	**t**	**CH$_2$-CO$_2^-$**	**33.63**
**Other Metabolites**				
**Choline**	**3.21**	**s**	**N-(CH$_3$)$_3$**	**56.55**
	3.51	t	CH$_2$	70.12
	4.07	t	CH$_2$	58.33
**Unknown Compounds**				
**U01 (Apigenin glycoside 1)**	**6.51**	**d**	**CH-6'**	**118.3**
	6.88	d	CH-5-3'	\
	6.79	d	CH-2'	\
**U02 (Apigenin glycoside 2)**	**6.59**	**d**	**CH-6'**	**118.08**
	7.22	d	CH-5-3'	\
	6.91	d	CH-2'	\
**U03 (2-Hydroxy-3-methylbutyric acid)**	**0.92**	**d**	\	18.76
	0.99	d	\	20.51
	2.19	m	\	44
**U04 (Octenoic acid)**	**0.87**	**t**	****	**37.15**
	1.28	m	\	28.33
	\	\	\	146.6
	\	\	\	76.19
	\	\	\	47.38
**U05 (Marlignan R)**	**5.96**	**st**	****	**127.77**
	5.61	bs	\	125.16
	6.41	d	\	136.01
	6.49	d	\	138.01
	3.04	bs	\	43.07
	2.09	st	\	22.49
	2.16	s	\	30.2

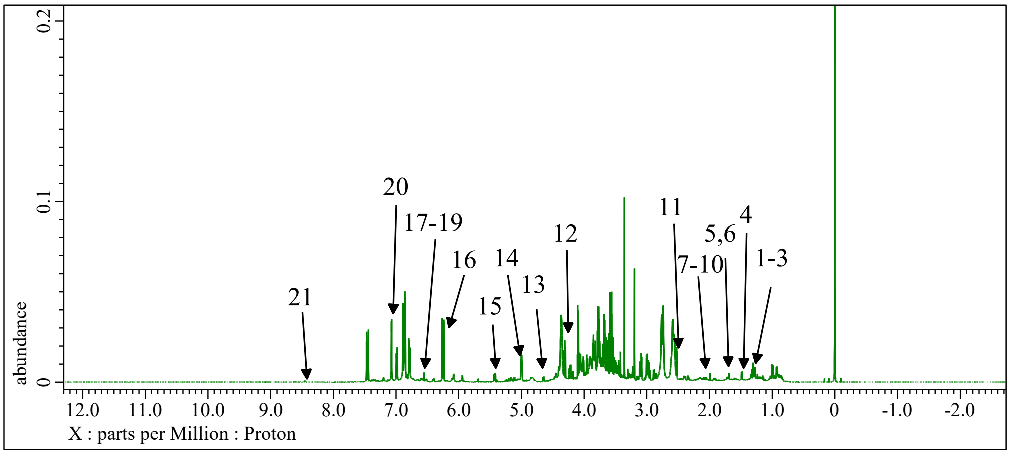

Supplementary Figure S5. ^{1}H spectrum of *C. aromaticus* leaves of BD-HAP extract. 1: U03 (2-Hydroxy-3-methylbutyric acid), 2: U04 (Octenoic acid), 3: threonine, 4: alanine, 5: GABA, 6: acetic acid, 7: glutamic acid, 8: glutamine, 9: malic acid, 10: asparagine, 11: choline, 12: fructose, 13: glucose, 14: raffinose, 15: sucrose, 16: caffeic acid, 17: U01 (Apigenin glycoside 1), 18: fumaric acid, 19: U02 (Apigenin glycoside 2), 20: rosmarinic acid, 21: formic acid.

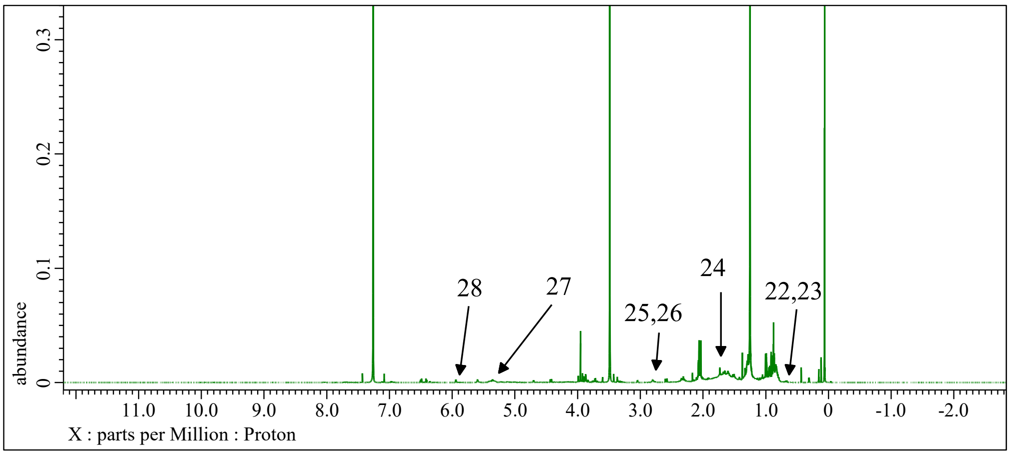

Supplementary Figure S6. ^{1}H spectrum of *C. aromaticus* leaves of BD-lipophilic extract. 22: β-sitosterol, 23: campesterol, 24: saturated fatty acids, 25: ω-6 FA, 26: ω-3 FA, 27: ω-9 FA, 28: U05 (Marlignan R).

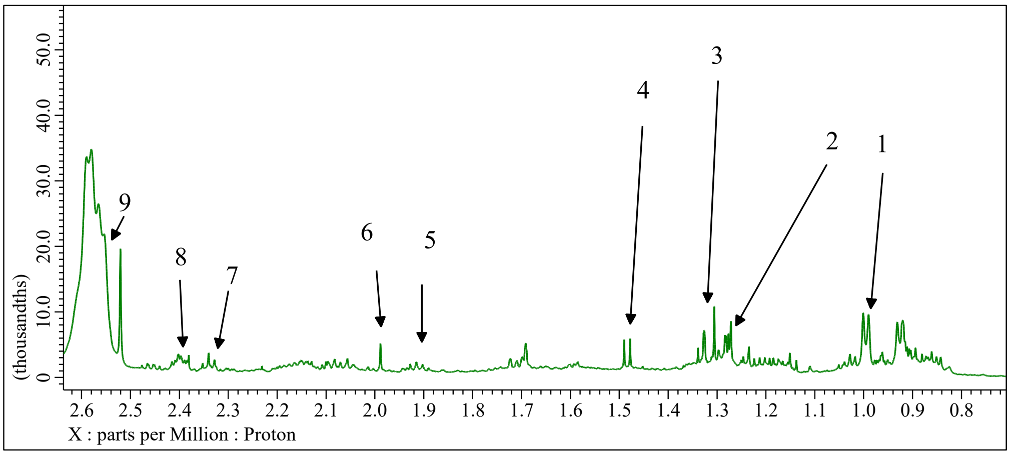

Supplementary Figure S7. ^{1}H spectrum of *C. aromaticus* leaves of BD-HAP extract, 2.6-0.8 ppm region. 1: U03 (2-Hydroxy-3-methylbutyric acid), 2: U04 (Octenoic acid), 3: threonine, 4: alanine, 5: GABA, 6: acetic acid, 7: glutamic acid, 8: glutamine, 9: malic acid.

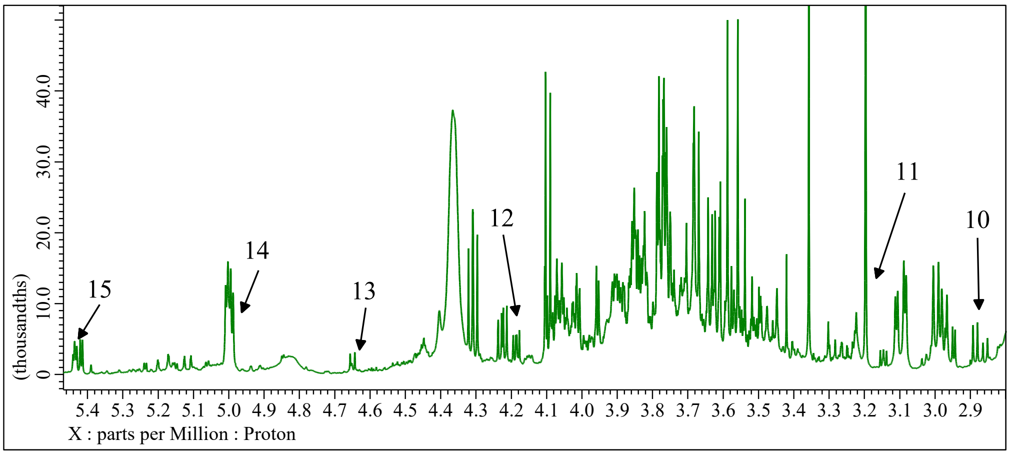

Supplementary Figure S8. ^{1}H spectrum of *C. aromaticus* leaves of BD-HAP extract, 5.4-2.9 ppm region. 10: asparagine, 11: choline, 12: fructose, 13: glucose, 14: raffinose, 15: sucrose.

.

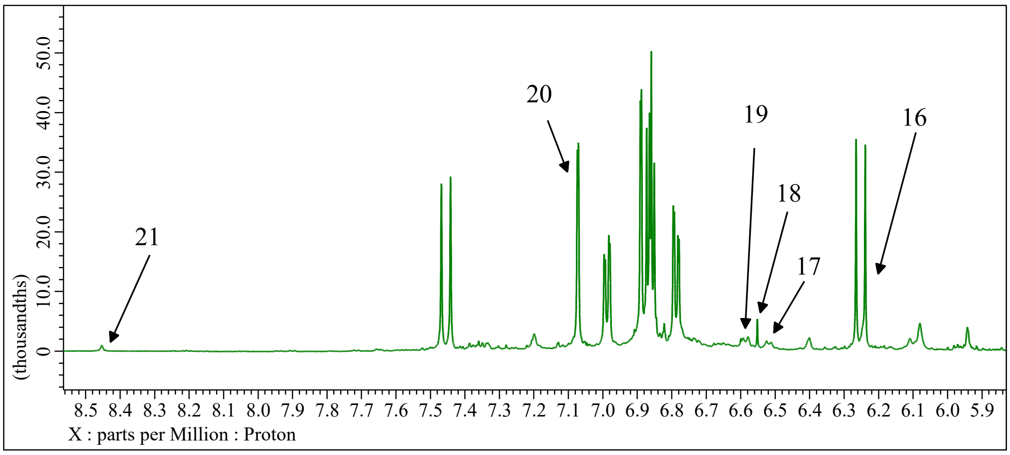

Supplementary Figure S9. ^{1}H spectrum of *C. aromaticus* leaves of BD-HAP extract, 5.9-8.5 ppm region. 16: caffeic acid, 17: U01 (Apigenin glycoside 1), 18: fumaric acid, 19: U02 (Apigenin glycoside 2), 20: rosmarinic acid, 21: formic acid.

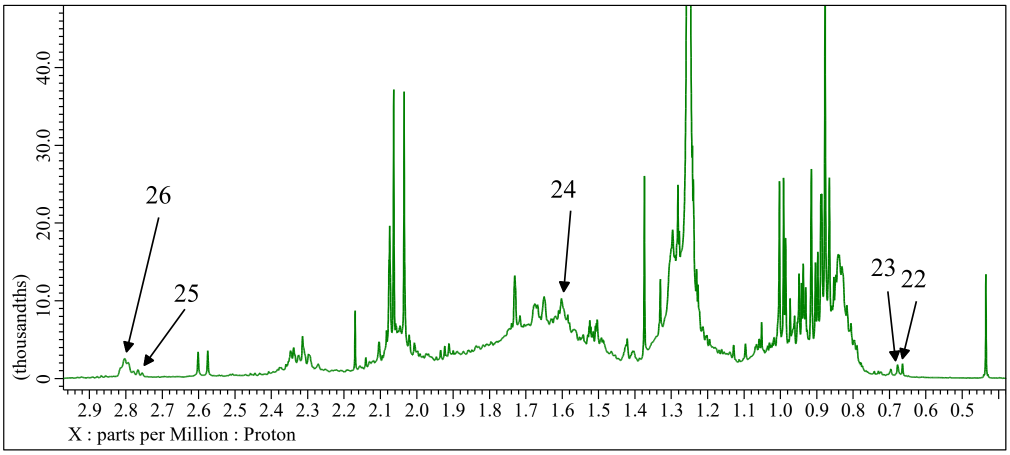

Supplementary Figure S10. ^{1}H spectrum of *C. aromaticus* leaves of BD-lipophilic extract, 0.5-2.9 ppm region. 22: β-sitosterol, 23: campesterol, 24: saturated fatty acids, 25: ω-6 FA , 26: ω-3 FA.

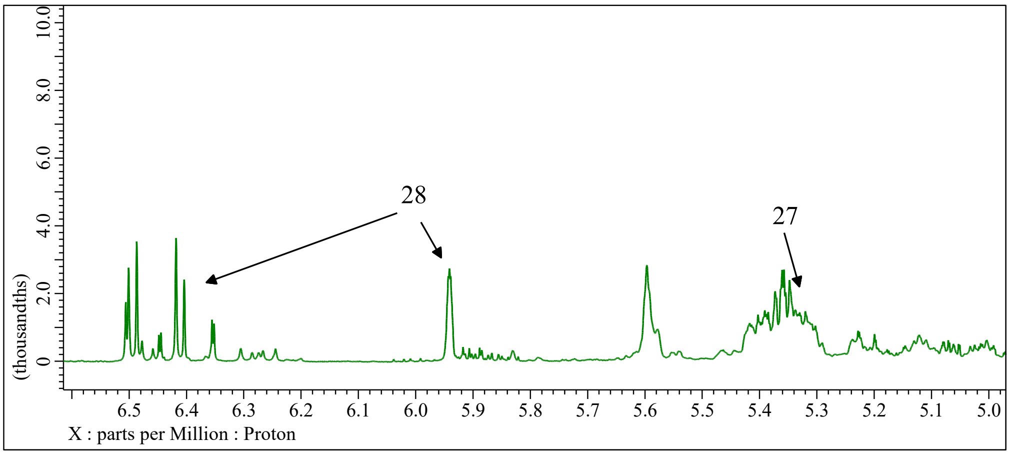

Supplementary Figure S11. ^{1}H spectrum of *C. aromaticus* leaves of BD-lipophilic extract, 5.0-6.5 ppm region. 27: ω-9 FA, 28: U05 (Marlignan R).

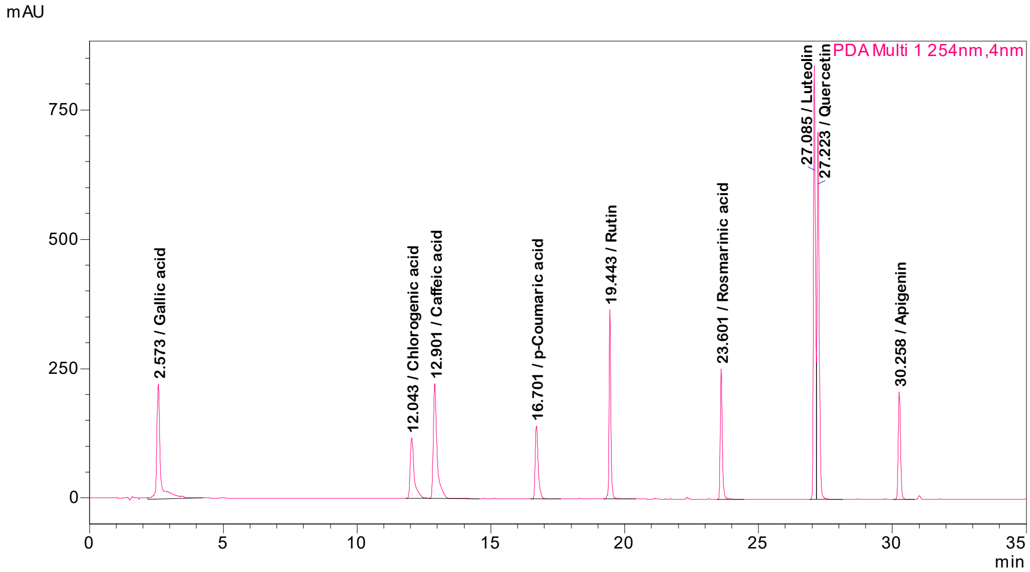

Supplementary Figure S12. HPLC chromatograms of standards with their retention times (min): gallic acid 2.57 (254 nm); chlorogenic acid 12,04 (254 nm); caffeic acid 12.90 (254 nm); rutin 19.44 (254 nm); rosmarinic acid 23.60 (254 nm); luteolin 27.08 (254 nm); quercetin 27.22 (254 nm); and apigenin 30.25 (254 nm).

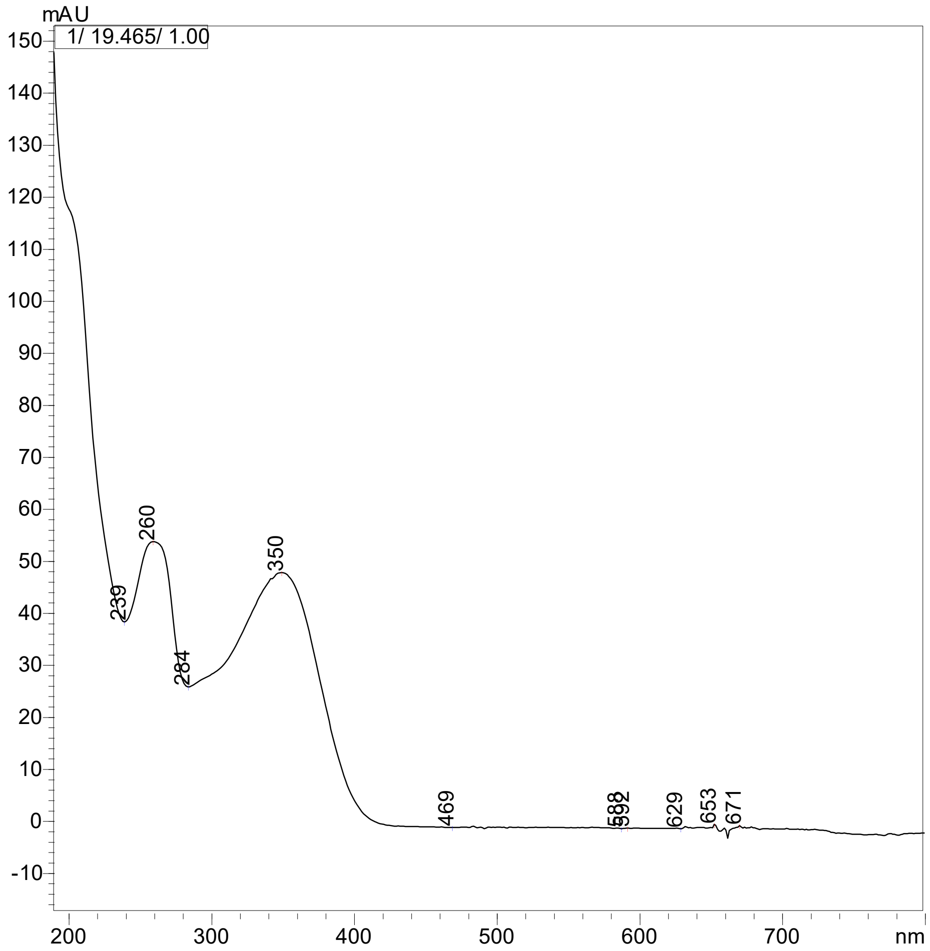
mAU
1/ 19.465/ 1.00
150
140
130
120
110
100
90
80
70
60
50
40
30
20
10
0
-10
260
350
239
284
469
629
653
671
200
300
400
500
600
700
nm

a.

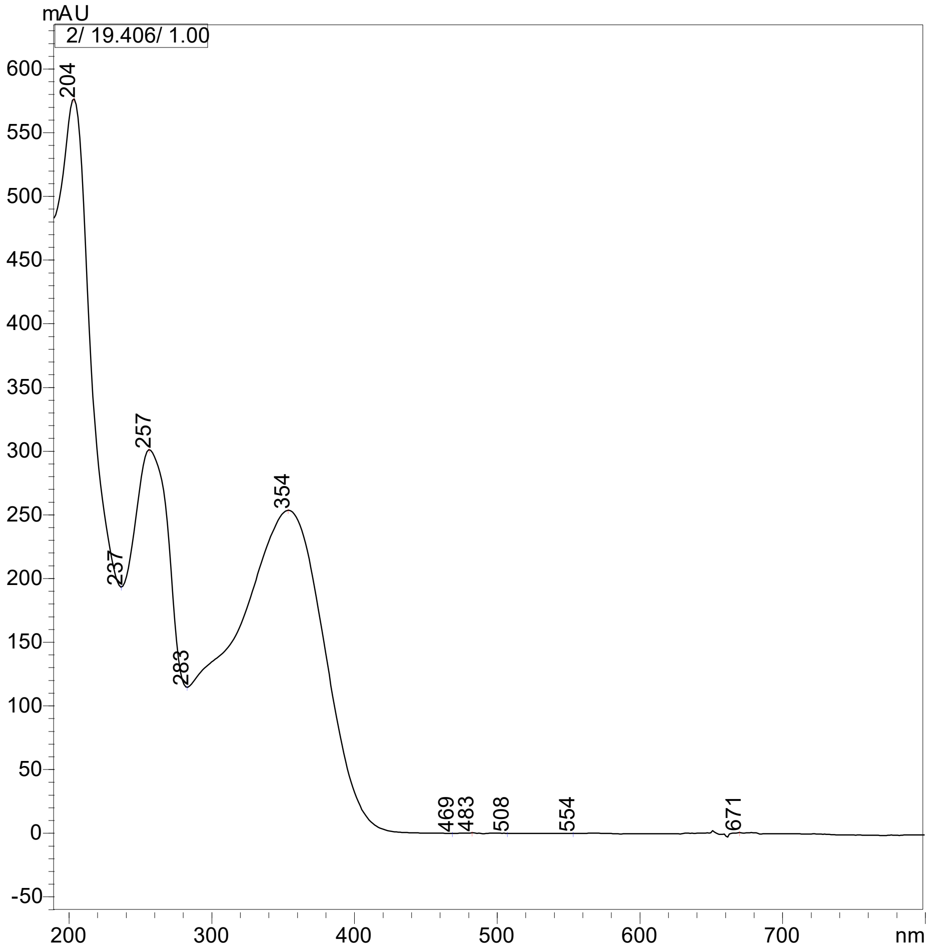
mAU
2/ 19.406/ 1.00
600
550
500
450
400
350
300
250
200
150
100
50
0
-50
204
257
354
237
283
469
483
508
554
671
200
300
400
500
600
700
nm

b.

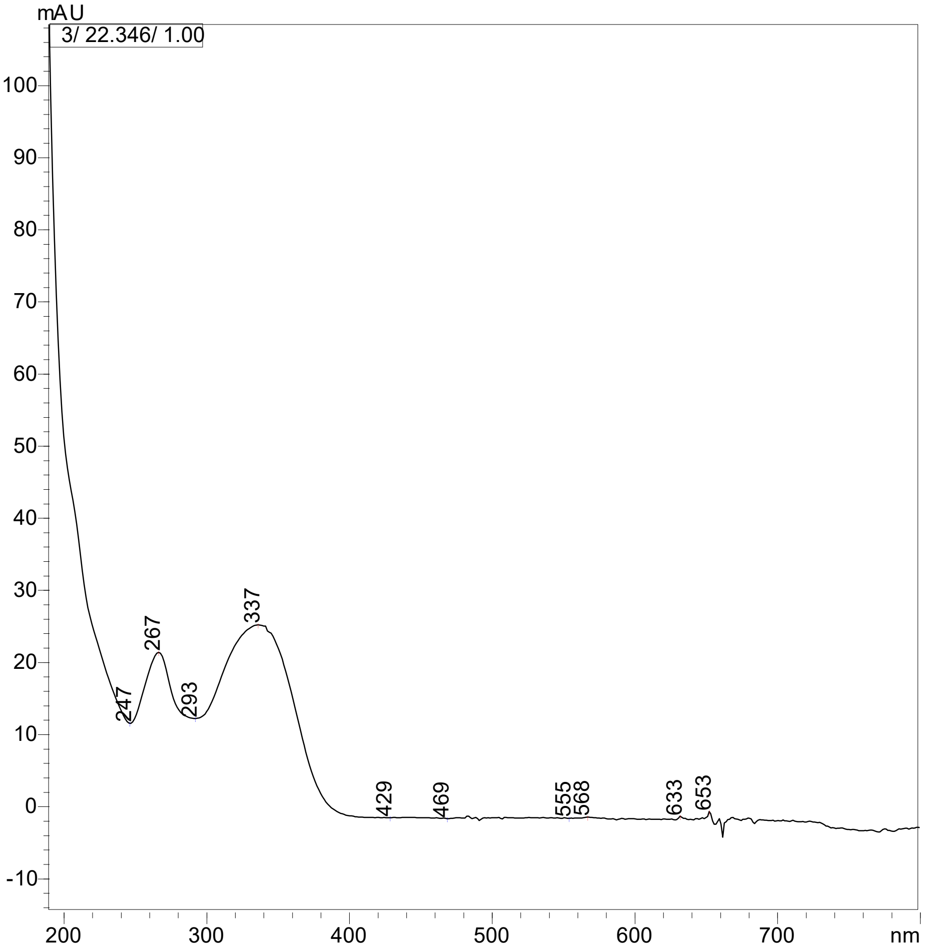
mAU
3/ 22.346/ 1.00
100
90
80
70
60
50
40
30
20
10
0
-10
247
267
293
337
429
469
555
568
633
653
200
300
400
500
600
700
nm

c.

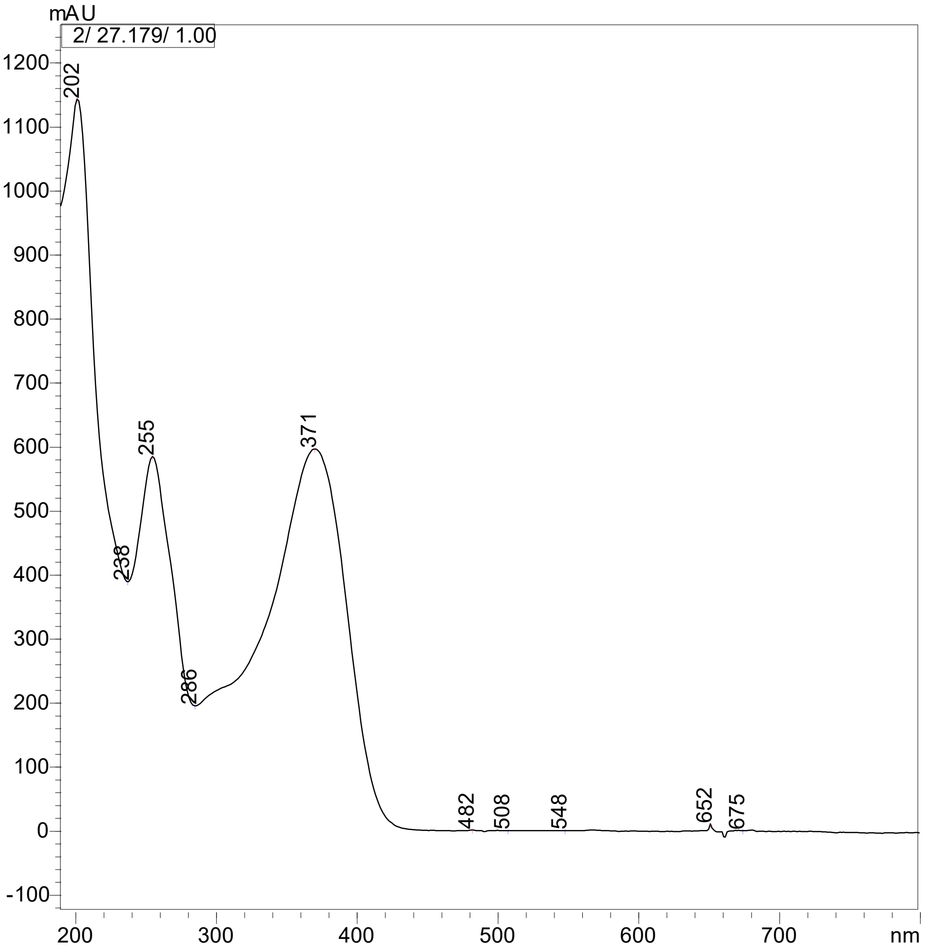
mAU
2/ 27.179/ 1.00
1200
1100
1000
900
800
700
600
500
400
300
200
100
0
-100
202
238
255
286
371
482
508
548
652
675
200
300
400
500
600
700
nm

d.

Supplementary Figure S13. UV–Vis spectra of (a) the unknown compound and the reference standards: (b) rutin, (c) apigenin, and (d) quercetin.
